# *TMPRSS2* gene polymorphism common in East Asians confers decreased COVID-19 susceptibility

**DOI:** 10.3389/fmicb.2022.943877

**Published:** 2022-11-30

**Authors:** Takeshi Sekiya, Yukino Ogura, Hirayasu Kai, Atsushi Kawaguchi, Shino Okawa, Mikako Hirohama, Takahiro Kuroki, Wataru Morii, Akira Hara, Yuji Hiramatsu, Shigemi Hitomi, Yasushi Kawakami, Yoshihiro Arakawa, Kazushi Maruo, Shigeru Chiba, Hiromichi Suzuki, Hiroshi Kojima, Hirokazu Tachikawa, Kunihiro Yamagata

**Affiliations:** ^1^Department of Infection Biology, Faculty of Medicine, University of Tsukuba, Tsukuba, Japan; ^2^Department of Nephrology, Faculty of Medicine, University of Tsukuba, Tsukuba, Japan; ^3^Transborder Medical Research Center, University of Tsukuba, Tsukuba, Japan; ^4^Microbiology Research Center for Sustainability, University of Tsukuba, Tsukuba, Japan; ^5^College of Biological Sciences, University of Tsukuba, Tsukuba, Japan; ^6^Graduate School of Comprehensive Human Sciences, University of Tsukuba, Tsukuba, Japan; ^7^Department of Otorhinolaryngology, Faculty of Medicine, University of Tsukuba, Tsukuba, Japan; ^8^Department of Cardiovascular Surgery, Faculty of Medicine, University of Tsukuba, Tsukuba, Japan; ^9^Department of Infectious Diseases, Faculty of Medicine, University of Tsukuba, Tsukuba, Japan; ^10^Department of Laboratory Medicine, Faculty of Medicine, University of Tsukuba, Tsukuba, Japan; ^11^Department of Medical Pharmacy, Faculty of Medicine, University of Tsukuba, Tsukuba, Japan; ^12^Department of Biostatistics, Faculty of Medicine, University of Tsukuba, Tsukuba, Japan; ^13^Department of Hematology, Faculty of Medicine, University of Tsukuba, Tsukuba, Japan; ^14^Ibaraki Clinical Education and Training Center, Faculty of Medicine, University of Tsukuba, Tsukuba, Japan; ^15^Division of Clinical Medicine, Department of Disaster and Community Psychiatry, Faculty of Medicine, University of Tsukuba, Tsukuba, Japan

**Keywords:** COVID-19, SARS-CoV-2, SNP, tmprss2, virus entry

## Abstract

COVID-19 has a wide range of clinical presentations, and the susceptibility to SARS-CoV-2 infection and the mortality rate also vary by region and ethnicity. Here, we found that rs12329760 in the *TMPRSS2* gene, a missense variant common in East Asian populations, contributes to protection against SARS-CoV-2 infection. TMPRSS2 is a protease responsible for SARS-CoV-2 entry and syncytium formation. rs12329760 (c.478G>A, p. V160M) was associated with a reduced risk of moderate symptoms. The enzymatic activity of Met^160^-TMPRSS2 was lower than that of Val^160^-TMPRSS2, and thus the viral entry and the syncytium formation of SARS-CoV-2 were impaired. Collectively, these results indicate that the genetic variation in *TMPRSS2*, which is common in East Asians, is one of the molecular determinants of COVID-19 susceptibility.

## Introduction

The COVID-19 pandemic caused by severe acute respiratory syndrome coronavirus 2 (SARS-CoV-2) has to date resulted in more than 5 million deaths worldwide. COVID-19 is associated mainly with respiratory symptoms and ranges from asymptomatic to fatal cases (Lai et al., 2020). Although the detailed mechanism remains unclear, several risk factors, such as old age, male sex, hypertension, and diabetes, have been reported to increase COVID-19 susceptibility and severity (Yang et al., 2020). It has also been observed that some European countries, such as Italy, the United Kingdom, and France, have higher mortality rates than those of East Asian countries including Japan, South Korea, and China, suggesting the involvement of other risk factors, such as environmental, cultural, and genetic differences, that are unrelated to the underlying disease (Iwasaki and Grubaugh, 2020; Yamamoto and Bauer, 2020).

To understand the host genetic contribution to COVID-19, a number of genome-wide association studies (GWAS) have been conducted to identify genetic variants involved in the development of COVID-19. The ABO blood group locus and a gene cluster on chromosome 3p21.23 were identified as involved in the severity of COVID-19 in Italian and Spanish populations (Ellinghaus et al., 2020). Genes related to host immunity and inflammation (*OAS1*, *OAS2*, *OAS3*, *TYK2*, *DPP9*, and *IFNAR2*) were also identified as critical determinants of the COVID-19 severity in United Kingdom patients (Pairo-Castineira et al., 2021). In a GWAS of COVID-19 patients in Japanese population, a single nucleotide polymorphism (SNP) in *DOCK2* gene was identified as a risk allele specific to East Asians but not to Europeans (Namkoong et al., 2022). However, SNPs that determine the lower COVID-19 susceptibility and mortality of East Asians have not yet been identified, possibly owing to the difficulty in comparing infected individuals with noninfected ones, including individuals who have not encountered the pathogen.

Transmembrane serine protease 2 (TMPRSS2) is responsible for the virus entry of SARS-CoV-2 by processing the viral spike (S) protein after viral attachment to angiotensin-converting enzyme 2 (ACE2; Hoffmann et al., 2020). TMPRSS2 is expressed in respiratory tracts and functions as a major host protease for the replication of not only SARS-CoV-2 but also HCoV-229E, SARS-CoV-1, MERS, and influenza A virus (Böttcher et al., 2006; Matsuyama et al., 2010; Bertram et al., 2013; Gierer et al., 2013). Previous reports showed that intronic SNPs in the *TMRPSS2* gene were associated with increased severity of influenza A virus and SARS-CoV-2 infections (Cheng et al., 2015; Schönfelder et al., 2021). These findings suggest that TMPRSS2 is one of the molecular determinants of susceptibility to SARS-CoV-2 infection and COVID-19 pathogenesis. In addition, several SNPs (rs423596, rs8134203, rs464431, rs2298662, rs2094881, rs75603675, rs456142, rs462574, and rs456298, rs12329760) in *TMPRSS2* showed a different allele frequency in Asians from that in other populations (Paniri et al., 2020). In Italian hospitalized COVID-19 patients, the allele frequency of rs12329760 in *TMPRSS2* is lower than that of controls in European GnomAD database (Latini et al., 2020), but not in German cohort cases (Schönfelder et al., 2021). In prostate cancer, the rs12329760 is associated with multiple copies of the *TMPRSS2-ERG* gene fusion leading to shorter survival and higher tumor recurrence (FitzGerald et al., 2008). Here we revealed that a missense variant rs12329760 (c.478G>A, p. V160M) in *TMPRSS2*, which changes the valine residue at amino acid position 160 to a methionine residue, contributed to protection against SARS-CoV-2 infection in medical care workers who had close contact with COVID-19 patients but did not become infected with the virus. The rs12329760 variant was common in East Asian populations as compared with European and African populations. Our results highlight the genetic difference in COVID-19 susceptibility among different ethnicities.

## Materials and methods

### Study design and participants

This study was conducted as a multicenter prospective observation pilot study with the University of Tsukuba, Division of Clinical Medicine, Faculty of Medicine, as a representative facility. The participants were recruited from areas in Ibaraki Prefecture, where many cases of nosocomial SARS-CoV-2 infection occurred among medical care workers.^1^ A detailed background survey was conducted of the medical care workers infected with SARS-CoV-2 (infected cases) and those who had close contact with COVID-19 patients but were not infected. Infected participants were defined as individuals with nasopharyngeal swabs or saliva that tested positive for SARS-CoV-2 on laboratory-based RT-PCR. Close contact was defined as direct contacts with COVID-19 patients during the course of providing medical care. The study participants comprised 25 infected participants and 139 noninfected close-contact participants. The participants were recruited to the study from 1 April 2020 to 19 February 2021. Additional genetic variant data of COVID-19 patients were provided by the Japan COVID-19 Task Force. All participants were not vaccinated.

### Data collection

Clinical information (including age, sex, body mass index, blood pressure, smoking, BCG vaccination, present illness, and exposure history) was obtained from the participants. Laboratory data, including complete blood count, blood sugar, coagulation profile, serum biochemical tests (total protein and albumin, electrolytes, renal and liver function, IgG, C-reactive protein), and urine, were measured at Tsukuba i-Laboratory LLP, Tsukuba, Japan. Serum angiotensin-converting enzyme was measured using ACE ELISA kits (AdipoGen Life Sciences; AG-45B-0023-KI01).

### Medical questionnaires

The questionnaire on the degree of contact included the following: The situation that comes to mind (yes or no); the medical care for COVID-19 patients: (1) None, (2) extremely light contact, (3) light contact (e.g., vital signs measurement), (4) tight contact (e.g., changing the patient’s position), (5) oral cavity and respiratory tract suction, (6) no recollection; and the rate of implementation (0, 25, 50, 75, and 100%) of personal protective measures, including hand-washing and wearing of a surgical mask, an N95 mask, eye protection, a medical gown, or medical gloves.

### Next-generation sequencing and variant analysis

Genomic DNA was extracted from whole blood samples obtained from the noninfected close contact group by use of MagExtractor genome (TOYOBO; NPK-101). The DNA libraries were prepared using a QIAseq Targeted DNA Custom Panel (Qiagen; 333,525) with region-specific primers for *TMPRSS2* according to the manufacturer’s protocol. The *TMPRSS2* gene locus was amplified as DNA fragments averaging 250 bp. Post-enriched libraries were quantified, pooled, and sequenced on the NextSeq500 sequencing platform (Illumina). Sequencing data were mapped to hg19 and variants were identified using CLC Genomics Workbench software (Qiagen). The linkage disequilibrium of the common SNPs within the *TMPRSS2* locus in East Asian populations was analyzed using LDlink.^2^

### Genotype imputation

The genotype data of rs12329760 of the asymptomatic-to-mild group, the moderate group, and the severe group were obtained from the Japan COVID-19 Task Force and analyzed by imputation. The genotyping data of Japan COVID-19 Task Force were obtained from Infinium Asian Screening Array and did not include rs12329760. Genotyped variant rs9305744, which is in linkage disequilibrium with rs12329760 (*r^2^* = 0.885), was used as a lead variant for genotype imputation using a population-specific reference panel of Japanese (1000 Genomes project EAS). Note that similar results were obtained for directly genotyped SNPs, rs9305744 (noninfected close-contact group vs. moderate-symptoms group *OR*: 0.553, 95% *CI* 0.3550–0.851, *p* = 0.008).

### Cytokine measurements

The concentrations of 48 cytokines and chemokines (sCD40L, EGF, Eotaxin, FGF-2, FLT3L, Fractalkine, G-CSF, GM-CSF, GROα/CXCL1, IFN-α2, IFN-γ, IL-1α, IL-1β, IL-1RA, IL-2, IL-3, IL-4, IL-5, IL-6, IL-7, IL-8, IL-9, IL-10, IL-12 p40, IL-12 p70, IL-13, IL-15, IL-17A, IL-17E/IL-25, IL-17F, IL-18, IL-22, IL-27, IP-10, MCP-1, MCP-3, M-CSF, MDC/CCL22, MIG/CXCL9, MIP-1α, MIP-1β, PDGF-AA, PDGF-AB/BB, TGF-α, TNF-α, TNF-β, VEGF-A, RANTES) were measured using the Bio-Plex Human Cytokine Screening Panel (Merck Millipore; HCYTA-60K-PX48) on a Luminex200 (Luminex Multiplexing Instrument, Merck Millipore) following the manufacturer’s instructions.

### Construction of cell lines constitutively expressing TMPRSS2 variants

A549 cells were purchased from the American Type Culture Collection and were maintained in Dulbecco’s minimal essential medium (DMEM) containing 10% fetal bovine serum and cultured at 37°C with 5% CO_2_. For the construction of plasmids expressing ACE2 and TMPRSS2, total RNA was reverse-transcribed as a template using the oligo(dT)_20_ primer, and the cDNAs were amplified with the primers 5′-TATGCGGCCGCCACCATGTCAAGCTCTTCCTGGCTCCTTC-3′ and 5′-TATGCGGCCGCCTAAAAGGAGGTCTGAACATCATCA-3′ for ACE2 and 5′-TATGAATTCCACCATGGCTTTGAACTCAGG-3′ and 5′-TATGCGGCCGCTTAGCCGTCTGCCCTCATT-3′ for TMPRSS2, and then cloned into the pCDH plasmid. The V160M substitution was introduced by the PCR-based site-directed mutagenesis using the primers 5′-CATCCTTCAGATGTACTCATCTCAG-3′ and 5′-CATCTGAAGGATGAAGTTTG-3′. The production of the lentivirus was carried out according to the manufacturer’s protocol. A549 cells were transduced with the lentivirus carrying ACE2. The A549 cells constitutively expressing ACE2 were further transduced with the lentiviruses expressing Val^160^-or Met^160^-TMPRSS2 (designated A549-ACE2-TMPRSS2-V160 and A549-ACE2-TMPRSS2-M160, respectively).

### Cell fusion assay

For the construction of plasmids expressing SARS-CoV-2 S protein, total RNA purified from SARS-CoV-2-infected cells was reverse-transcribed as a template using the oligo(dT)_20_ primer, and the cDNA was amplified with the primers 5′-CGGTATCGATAAGCTTGATGCCACCATGTTTGTTTTTCTTGTTTTATTGCCACTAG-3′ and 5′-CCCGGGCTGCAGGAATTCGATTTATGTGTAATGTAATTTGACTCCTTTGAGCACTGGCTCAGAGTCGTCTTCATCAAATTTGCAGCAGGATCCACAAGAACAAC-3′, and then cloned into pCAGGS-p7 plasmid. For the transient transfection, 1 × 10^4^ HEK293T cells were inoculated on a 12-well plate and transfected with plasmids expressing S protein and GFP using Lipofectamine 2000 (Life Technologies; #11668019) according to the manufacturer’s instruction. After incubating for 5 h at 37°C, 1 × 10^5^ of either A549-ACE2-TMPRSS2-V160 or M160 cells were added and further incubated for 24 h. The cells were then fixed with 2% PFA for 15 min and stained with Hoechst33342. To quantify the level of syncytium formation, the number of GFP-positive cells containing more than two nuclei was counted using ImageJ software. The ratio of the cells showing syncytium formation to total cells was calculated.

### Quantitative RT-PCR

Total RNAs were isolated from A549, A549-ACE2-TMPRSS2-V160, and A549-ACE2-TMPRSS2-M160 cells, respectively. cDNA was prepared from 1 μg of total RNAs by use of ReverTraAce (TOYOBO; TRT-101) with oligo(dT)_20_ primer. Real-time PCR was carried out using SYBR Green Realtime PCR Master Mix-Plus (TOYOBO; QPK-211) in the Thermal Cycler Dice Real-Time PCR system (TaKaRa). The Cp values were calculated by the second derivative maximum method using a standard curve. The primer sequences used in this study were 5′-ATGGCATTGGACGGCATTTG-3′ and 5′-TGTTCTGGCTGCATCAT-3′ for *TMPRSS2* and 5′-AACGGCTACCACATCCAAGG-3′ and 5′-GGGAGTGGGTAATTTGCGC-3′ for 18S rRNA.

### Antibodies

Rabbit anti-TMPRSS2 (Abcam; ab242384) and mouse anti-β-actin (SIGMA; A5441) antibodies were purchased. Mouse anti-Spike antibody was kindly provided by Dr. Kensaku Murano (Keio University) (Guo et al., 2021).

### Single-recognition *in situ* proximity ligation assay (PLA)

Cells were infected with SARS-CoV-2 at an MOI of 100 on ice for 30 min. After the virus adsorption, the cells were washed twice with ice-cold DMEM and then further incubated at 37°C for 5, 10, and 15 min. The cells were then fixed with 4% PFA and incubated with 1% milk for 30 min. The cells were incubated with 10 ng/μl mouse anti-Spike antibody for 1 h and fixed again with 4% PFA. PLA was carried out using a Duolink *In Situ* PLA kit (Olink Bioscience) with anti-mouse PLUS (DUO92001) and anti-mouse MINUS probes (DUO92004) according to the manufacturer’s protocol. Briefly, the cells were incubated with anti-mouse PLUS and anti-mouse MINUS probes for 1.5 h at room temperature, and then washed with Wash Buffer A. The cells were further incubated with PLA ligase for 45 min at 37°C. After washing, the cells were subjected to the amplification reaction using the polymerase, which is supplied in the kit, for 2.5 h at 37°C. After final washing using Wash Buffer B, the cells were stained with 4,6-diamidino-2-phenylindole (DAPI) for 10 min. The number of PLA signals was measured using IMARIS software (Carl Zeiss).

### Statistics analysis

Continuous variables were expressed as either medians with interquartile ranges (IQRs) or means with standard deviations (SDs). Categorical variables were expressed as numbers and percentages. Significant differences were determined using the Mann–Whitney *U*-test, the Chi-square test, or 1-way ANOVA with the Tukey test for continuous variables and the Fisher exact test for categorical variables using GraphPad Prism software (version 9.0.2). The statistical power was calculated by *R* statistical software (version 3.6.0). The Hardy–Weinberg Equilibrium (HWE) was calculated by Chi-square test. The multivariate logistic regression analysis with Wald test was performed to examine the genetic association of rs12329760 between the asymptomatic-to-mild and moderate groups to adjust for age and gender using *R* statistical software (version 3.6.0). n/s, not significant. **p < 0.01, *p < 0.05. The *Q* value based on the false discovery rate (FDR) was calculated to adjust for multiple comparisons using the Benjamini-Hochberg test. In the present study, statistical significance was defined as a *q* < 0.05.

## Results

### Clinical characteristics of study participants and the degree of contact to the COVID-19 patients

A total of 164 medical care workers who had close contact with COVID-19 patients were enrolled in the study; 25 of the medical care workers became infected with SARS-CoV-2, while the remaining one hundred 39 did not (Supplementary Table S1). All the participants were of Japanese ethnicity. No remarkable differences were found between the infected and noninfected participants in terms of prevalence of underlying diseases, BCG vaccination history, smoking, and body mass index. The low number of participants with underlying diseases may be attributable to the fact that most of the participants were relatively young (Supplementary Table S1). Blood and urine test results showed slightly higher total protein and IgG levels as well as higher estimated glomerular filtration rates in the infected participants (Supplementary Table S2) but no other differences between the infected and noninfected participants. The normal physiological levels of serum cytokines and chemokines were also examined to assess the presence of underlying inflammatory diseases (Supplementary Table S3). But, there were no cytokines or chemokines apparently induced in infected participants compared to noninfected participants. In contrast, in terms of medical practice, tight contact with COVID-19 patients, such as *via* repositioning and suctioning of the oral cavity and respiratory tract, was significantly higher in the infected participants than in the noninfected participants (Supplementary Table S4), with about 28.1% of the noninfected participants having carried out similar medical practices. No significant differences were found between the infected and noninfected participants in terms of the rates of implementation of personal protective measures such as hand-washing and wearing of masks, medical gowns, and medical gloves (Supplementary Table S5).

### Identification of genetic variants in *TMPRSS2* gene

**Figure 1:**
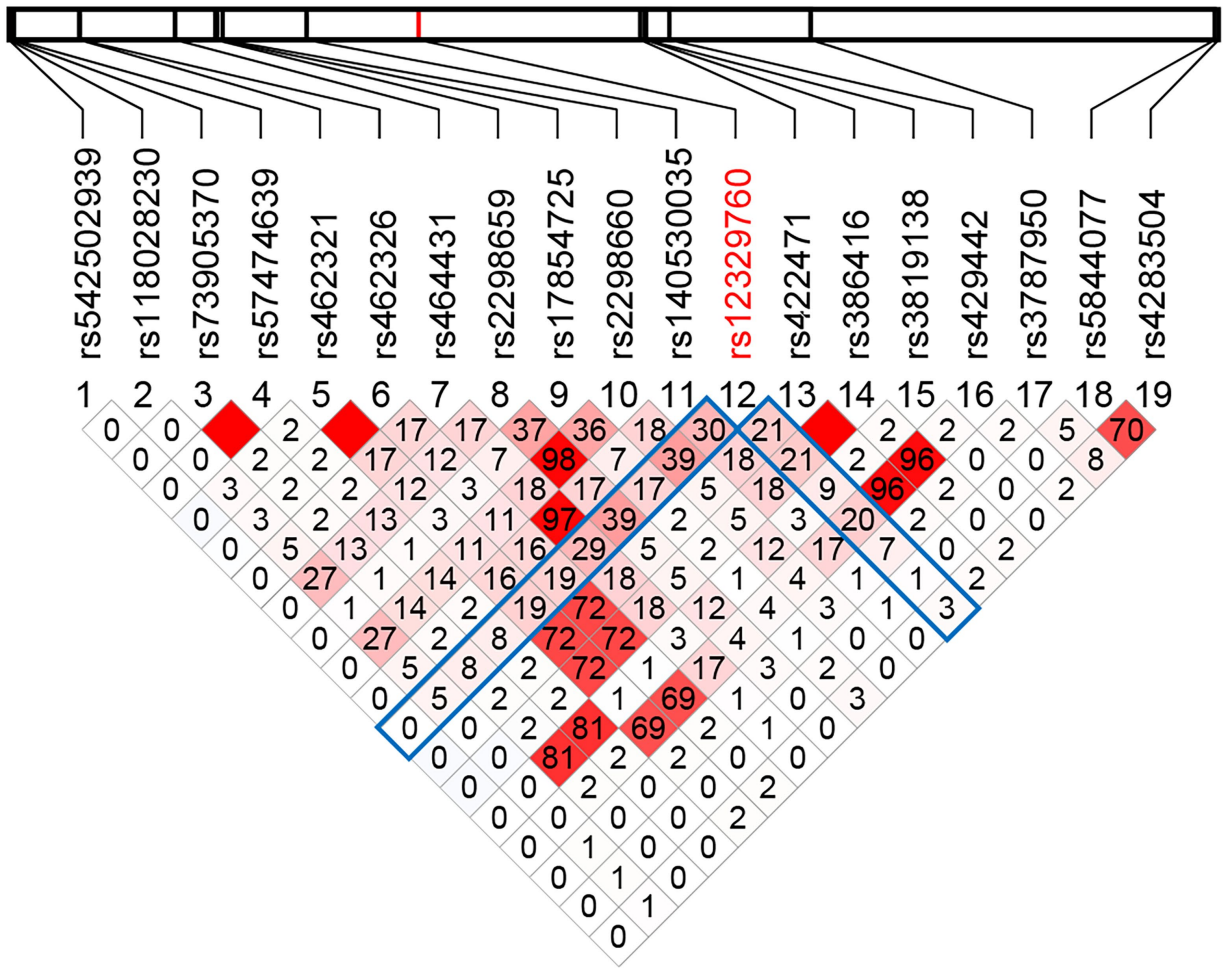
The linkage disequilibrium of the 19 common SNPs within the *TMPRSS2* locus in East Asian populations. The linkage disequilibrium of the 19 common SNPs within the *TMPRSS2* locus in East Asian populations was analyzed using the genotype data from 1,000 Genomes Project. The physical position of each SNP is shown above the plot. The values of *r*^2^ × 100 are shown in the boxes. The square without number corresponds to *r*^2^ = 1.0. The red scale represents the LD status between each pair of SNPs (*r*^2^ = 1 red; *r*^2^ = 0 white).

We next examined the associations of SNPs in the *TMPRSS2* gene with susceptibility to SARS-CoV-2 infection. The variant data identified in the noninfected close-contact group were compared with those in 1527 infected patients enrolled in the Japan COVID-19 Task Force.^3^ The infected cases were classified according to their clinical symptoms, ranging from asymptomatic to mild (symptomatic but not on oxygen support or artificial respiration), moderate (on oxygen support or artificial respiration), and severe (intensive-care unit hospitalization; Table 1). We found 74 SNPs in the *TMPRSS2* gene by targeted sequencing. Of those 74 SNPs, the allele frequencies of 19 SNPs were above 5% in the noninfected close contacts, but the others were rare SNPs with allele frequencies below 5%. Four (rs12329760, rs2298659, rs17854725, and rs3787950) of the 19 SNPs were located in the exons of *TMPRSS2*, and only rs12329760 C>T was a missense variant; this variant causes the substitution of valine at amino acid position 160 to methionine. The rs12329760 and other identified SNPs within the *TMPRSS2* locus were not in linkage disequilibrium (Figure 1). The minor allele (allele T) of the rs12329760 occupied 38.8% in the noninfected close-contact group, 38.3% in the asymptomatic-to-mild group, 33.4% in the moderate group, and 35.9% in the severe group (Table 2). Frequencies of three genotypes C/C, C/T, and T/T of rs12329760 were 39.0, 47.6, and 13.4%, respectively, in all participants (Table 2). The number of T/T individuals was lower than that of C/C and C/T individuals. The Hardy–Weinberg equilibrium was calculated for total sample set and for each group of the set, and the result showed that the frequencies of these genotypes were in equilibrium (*p* > 0.05).

**Table 1:** Characteristics of noninfected close-contact participants in this study and of infected groups enrolled in the Japan COVID-19 Task Force classified by severity of COVID-19.

Age, median (IQR), years	Noninfected close contact (*n* = 139)		Asympt to mild (*n* = 1,063)		Moderate (*n* = 283)		Severe (*n* = 181)	
	40	(31–48)	40	(29–53)	54	(46–59)	58	(51–62)
Sex, *n* (%)								
Male	43	(30.9%)	627	(59.0%)	231	(81.6%)	146	(80.7%)
Female	96	(69.1%)	436	(41.0%)	52	(18.4%)	35	(19.3%)

**Table 2:** Contingency tables for *TMPRSS2* genotype in the noninfected close-contact group and the infected groups classified by severity of COVID-19.

	Close contact (*n* = 139)	Asympt to Mild (*n* = 1,063)	Moderate (*n* = 283)	Severe (*n* = 181)	Total (*n* = 1,666)
Genotype *n* (%)					
C/C (Val/Val)	49 (35.3)	400 (37.6)	129 (45.6)	71 (39.2)	649 (39.0)
C/T (Val/Met)	72 (51.8)	512 (48.2)	119 (42.0)	90 (49.7)	793 (47.6)
T/T (Met/Met)	18 (12.9)	151 (14.2)	35 (12.4)	20 (11.0)	224 (13.4)
Alleles *n* (%)					
C allele	170 (61.2)	1,312 (61.7)	377 (66.6)	232 (64.1)	2091 (62.8)
T allele	108 (38.8)	814 (38.3)	189 (33.4)	130 (35.9)	1,241 (37.2)
HW equilibrium					
*p*-value	0.56	0.84	0.65	0.31	0.76

**Figure 2:**
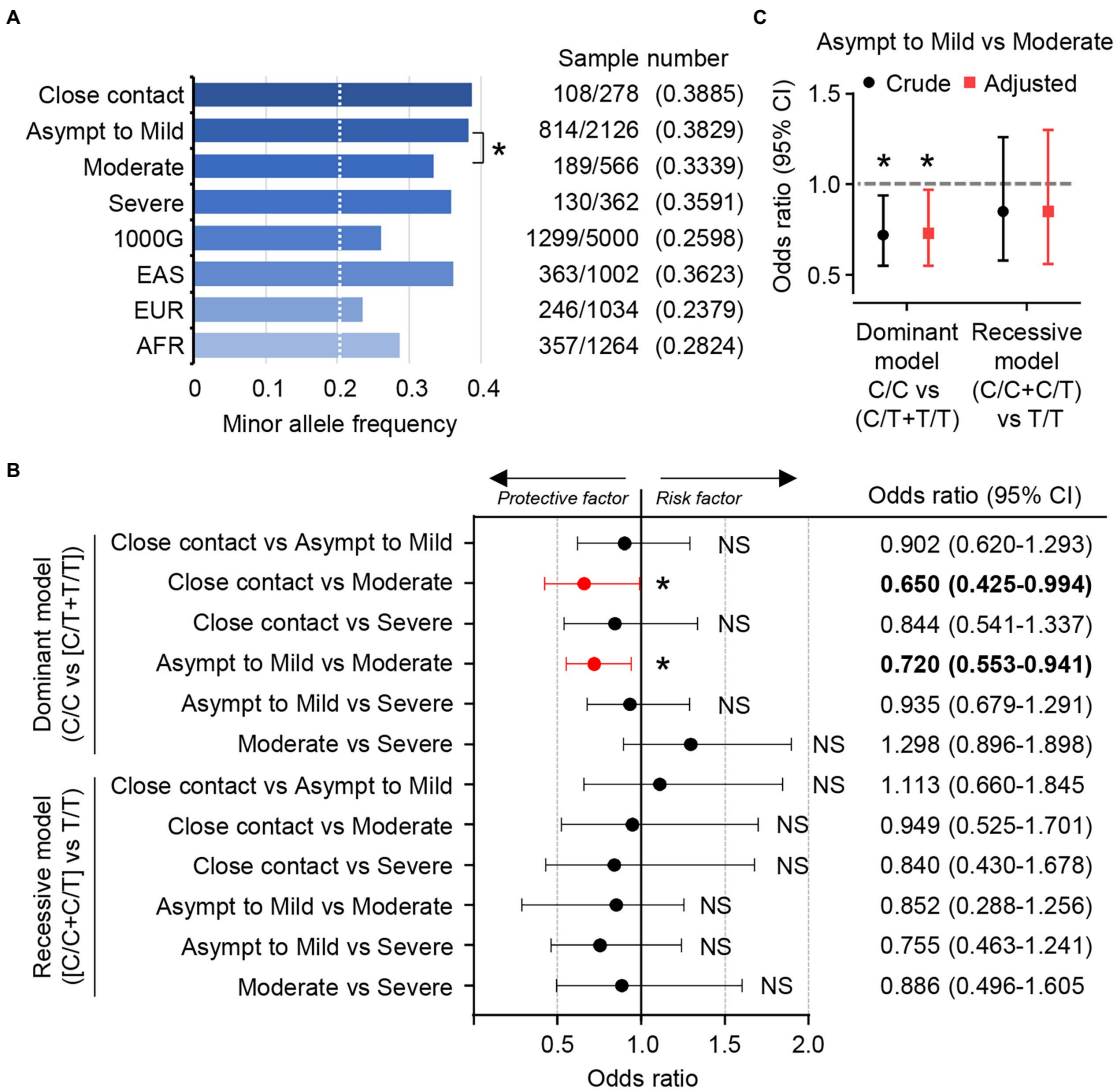
rs12329760 in the *TMPRSS2* gene is associated with susceptibility to SARS-CoV-2. **(A)** Minor allele frequency for rs12329760 from this study and the 1000 Genomes database. 1000G, all populations in the database; EAS, East Asian; EUR, European; AFR, African. *p < 0.05, **p < 0.01; Chi-square test. **(B)** Odds ratios for *TMPRSS2* rs12329760 in the noninfected close contact group and infected groups classified according to the severity of COVID-19 represented in the minor allele dominant model (C/C vs. [C/T + T/T]) and the minor allele recessive model ([C/C + C/T] vs. T/T). NS; not significant, *p < 0.05; Fisher exact test (2-tailed). **(C)** Odds ratios for *TMPRSS2* rs12329760 in the asymptomatic-to-mild group and the moderate group represented in the minor allele dominant model (C/C vs. [C/T + T/T]) and the recessive model ([C/C + C/T] vs. T/T) examined by the multivariate logistic regression model adjusting for age and gender. NS, not significant; *p < 0.05; Wald’s test.

The MAF of the T allele in *TMPRSS2* was highly enriched in our cohort (0.388) and in East Asians (0.362) as compared with in Europeans (0.236) and Africans (0.287) as reported in the 1000G database, a database of population genetic variations (Figure 2A).We also found that the MAF of the T allele in the patients with moderate COVID-19 symptoms tended to be lower than that in the noninfected close-contact group and in the patients with asymptomatic to mild symptoms (0.334 vs. 0.388 or 0.383; Figure 2A).

We next calculated the odds ratios and confidence intervals using the Baptista-pike method and *p*-value was obtained using two-sided Fisher’s exact test. In the dominant model (C/C vs. [C/T + T/T]), carriers of the T allele tended to be a reduced risk of moderate COVID-19 symptoms (noninfected close-contact group vs. moderate-symptoms group, *OR*: 0.650, 95% *CI* 0.425–0.994, *p* = 0.047, *q* = 0.5137; Figure 2B). A similar result was obtained when the patients with asymptomatic to mild symptoms were compared with those with moderate symptoms (*OR*: 0.720, 95% *CI* 0.553–0.941, *p* = 0.017, *q* = 0.1980; Figure 2B). However, the T allele in *TMPRSS2* had no protective effect against development of severe COVID-19 symptoms. In contrast to the dominant model, no differences were found in the recessive model ([C/C + C/T] vs. T/T).

The median ages of the moderate and severe groups were 54 and 58 years, respectively, while it was 40 years in noninfected close contact and asymptomatic-to-mild groups (Table 1). Further, more than 80% of infected patients who presented with moderate or severe symptoms were male, indicating the sex difference in the severity of COVID-19, as previously reported (Scully et al., 2020). Next, the multivariate logistic regression analysis was performed to examine the genetic association of rs12329760 between the asymptomatic-to-mild and moderate groups to adjust for age and gender (Figure 2C). We found that the adjusted odds ratio for age and gender was 0.73 (95% *CI* 0.56–0.97) and the crude odds ratio was 0.72 (95% *CI* 0.55–0.94) in the dominant model. This suggests that age and gender differences are not confounding factors of the susceptibility to SARS-CoV-2 as determined by rs12329760.

### TMPRSS2-mediated viral entry is impaired by V160M substitution

**Figure 3:**
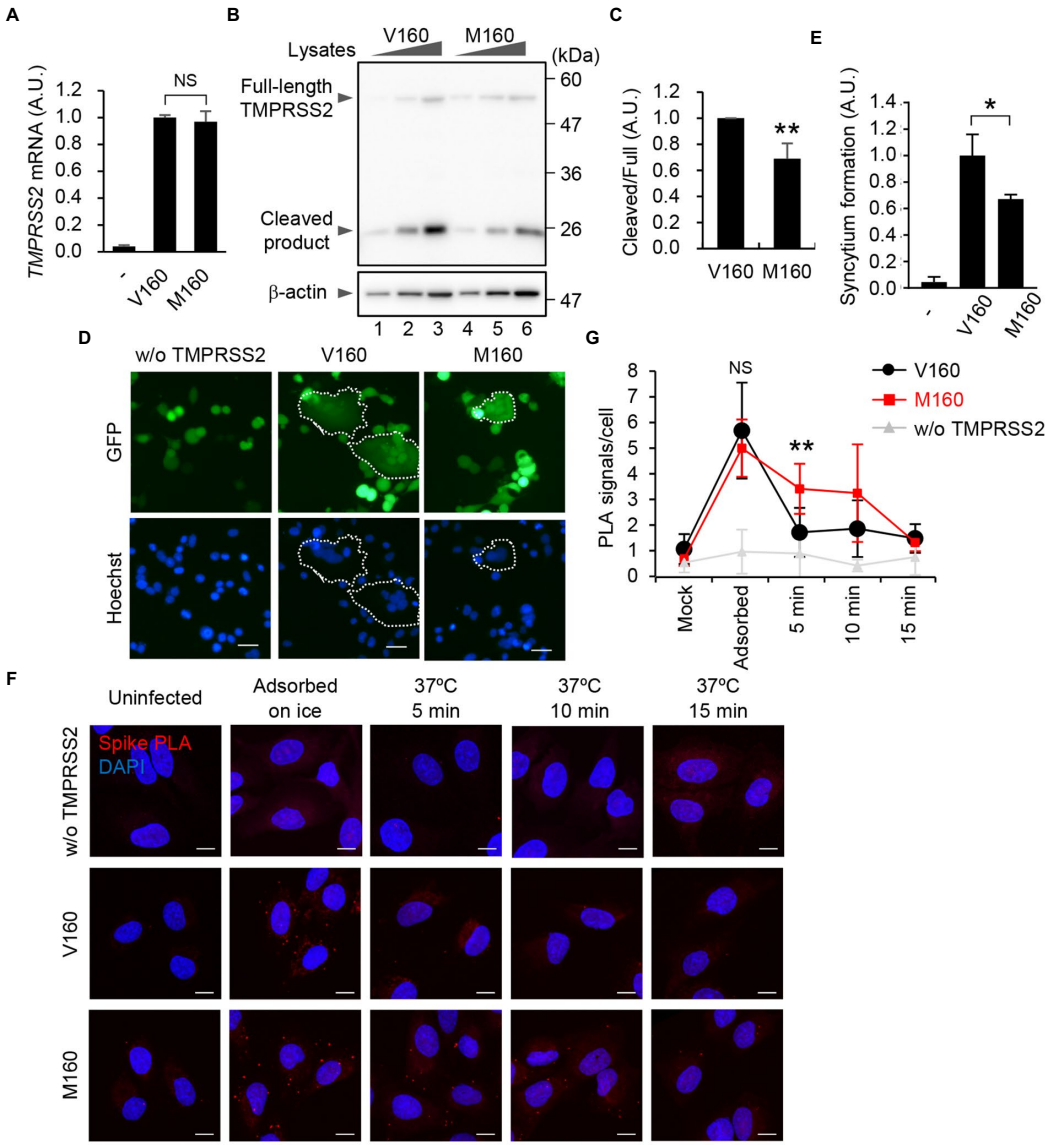
V160M substitution of TMPRSS2 delays the viral entry of SARS-CoV-2. **(A)** Level of *TMPRSS2* mRNA. Total RNAs isolated from A549, A549-ACE2-TMPRSS2-V160 (V160), or A549-ACE2-TMPRSS2-M160 (M160) cells were subjected to quantitative RT-PCR with primers specific for *TMPRSS2* mRNA. NS; not significant, Student’s *t* test. Means ± SDs from 3 independent experiments. **(B)** Expression level of TMPRSS2 protein. The cell lysates obtained from A549-ACE2-TMPRSS2-V160 or M160 cells (5 × 10^3^, 1 × 10^4^, and 2 × 10^4^ cells) were analyzed using SDS-PAGE followed by western blot assays with anti-TMPRSS2 and anti-β-actin antibodies. Data are representative of 3 independent experiments. **(C)** The quantitative data of panel B are shown. **p < 0.01; 2-tailed Student’s *t* test. Means ± SDs from 3 independent experiments. **(D)** HEK293T cells transfected with plasmids expressing spike and GFP (1 × 10^4^ cells) were co-cultured with A549-ACE2-TMPRSS2-V160 or M160 cells (1 × 10^5^ cells). At 24 h post-transfection, the cells were fixed in 2% PFA and stained with Hoechst33342 (Blue). The dotted circle encloses the syncytia. Scale bar, 40 μm. Data are representatives of 3 independent experiments. **(E)** The quantitative data of panel D are shown with means ± SDs. The number of syncytia was counted and normalized with total GFP-positive cells (*N* > 400). *p < 0.05, **p < 0.01; 2-tailed Student’s *t*-test. **(F)** A549, A549-ACE2-TMPRSS2-V160 or M160 cells were infected with SARS-CoV-2 at an MOI of 100 on ice for 30 min. The cells were further incubated at 37°C for 5, 10, and 15 min. After fixation, the cells were subjected to single-recognition *in situ* PLA with anti-Spike antibody. Scale bar, 10 μm. Data are representative of 3 independent experiments. **(G)** The number of PLA signals per cells is shown with means ± SDs (*n* > 30). NS; not significant, **p < 0.01; 1-way ANOVA with the Tukey test.

TMPRSS2 is classified as a type II transmembrane protein (TTSP) consisting of an N-terminal cytoplasmic domain, a transmembrane domain, an LDL receptor class A (LDLR-A) domain, a scavenger receptor cysteine-rich (SRCR) domain, and a C-terminal extracellular serine protease (SP) domain. It is known that TTSP family proteins are expressed as zymogens and activated by proteolytic cleavage (Bugge et al., 2009). TMPRSS2 is processed by self-cleavage between the SRCR domain and the protease domain to generate an active protease. To address the impact of the rs12329760 on SARS-CoV-2 infection *in vitro*, we established A549 cells exogenously expressing the *TMPRSS2* gene carrying Val^160^ (A549-ACE2-TMPRSS2-V160) or Met^160^ (A549-ACE2-TMPRSS2-M160; Figure 3). Our A549 cells expressed negligible endogenous *TMPRSS2* mRNA (Figure 3A). In the cell lines we constructed, the mRNA level of M160 was comparable to that of V160 (Figure 3A). The amount of full-length TMPRSS2 protein was not altered between V160 and M160, although the amount of cleaved product of M160 was reduced to 68% of that of V160 (Figures 3B,C), suggesting that the proteolytic activation of TMPRSS2 was impaired by the V160M substitution.

Viral S protein is cleaved into S1 and S2 subunits by the endopeptidase furin. Then, the S2 subunit is further processed into S2′ by TMPRSS2 to activate its membrane fusion activity for the virus entry and the cell–cell fusion, producing multinuclear giant cells, known as syncytia (Yu et al., 2022). To examine the proteolytic activation of S protein by TMPRSS2 carrying V160 or M160, HEK293T cells were transfected with plasmids expressing S protein and GFP and then co-cultured with A549-ACE2-TMPRSS2-V160 or M160 cells (Figures 3D,E). At 24 h post-transfection, spike-driven syncytium formation was observed in A549 cells expressing TMPRSS2, but not in cells without TMPRSS2 expression (Figures 3D,E), suggesting that the syncytium formation is dependent on the expression of TMPRSS2. Further, similar to the proteolytic activation level of TMPRSS2 (Figures 3B,C), the number of syncytium formation of M160 was also reduced to 65% of that of V160 (Figure 3E).

We next examined the viral entry of SARS-CoV-2 into A549-ACE2-TMPRSS2-V160 or M160 cells by detecting the viral particles on the plasma membrane using single-recognition *in situ* PLA with anti-Spike antibody. We could visualize the single viral particles, which are below the optical microscope resolution limit, by enhancing with single-recognition *in situ* PLA (Figure 3F). A549-ACE2-TMPRSS2-V160 or M160 cells were infected with SARS-CoV-2 at an MOI of 100 on ice for 30 min. After virus adsorption, the cells were further incubated at 37°C for 5, 10, and 15 min to allow the entry of viral particles. We found that the number of viral particles attached to the plasma membrane was comparable between A549-ACE2-TMPRSS2-V160 and M160 cells after incubation on ice for 30 min (Figures 3F,G). The adsorbed viral particles were rapidly internalized from the plasma membrane within 5 min in A549-ACE2-TMPRSS2-V160 cells after incubating at 37°C, and the number of viral particles on the plasma membrane reached to the background level similar to A549 cells not expressing TMPRSS2 (Figure 3G). In contrast, the viral particles were observed in A549-ACE2-TMPRSS2-M160 cells even 5 to 10 min after incubation (Figures 3F,G). These findings suggest that the V160M substitution of TMPRSS2 delays the viral entry of SARS-CoV-2, possibly owing to the impaired proteolytic activity of TMPRSS2.

## Discussion

Although risk factors for increased susceptibility to SARS-CoV-2 infection and disease severity have been identified in part, few studies have been conducted to investigate the genetic factors associated with the low incidence of and mortality from COVID-19 in East Asian populations. Many medical care workers are at risk of becoming infected with SARS-CoV-2 due to close contact with the infected patients through their medical practice. We therefore evaluated the clinical information, degree of contact with infected patients, serum cytokines/chemokines, and genetic variants of human genes related to SARS-CoV-2 infection in medical care workers who had close contact with infected patients but were themselves not infected. This study revealed that rs12329760 in the *TMPRSS2* gene, a missense variant common in East Asian populations, contributes to protection against SARS-CoV-2 infection and development of COVID-19. rs12329760 (c.478G>A, p. V160M) was associated with a reduced risk of moderate symptoms. Medical practices such as repositioning and respiratory tract suction increased the infection risk, but our study revealed that the genetic variants in the *TMPRSS2* gene contributed to increased susceptibility to SARS-CoV-2 infection, although the statistical power was not optimal due to the limited number of participants (the dominant model; 0.5349 for the close contact noninfected group vs. the moderate group and 0.6714 for the asymptomatic-to-mild group vs. the moderate group). The trend was statistically observed, but no significant difference was obtained after statistical correction of FDRs for the odds ratios (Figure 2B). Further analyses in different populations are required to replicate our findings.

The ectodomain of TMPRSS2 is composed of the LDLR-A domain, the SRCR domain, and the SP domain. The LDLR-A domain is relatively flexible and serves as a link between the protease and the plasma membrane. The SRCR domain has a globular fold consisting of curved β-sheets wrapped around an α-helix and tethers the SP domain by a disulfide bond (Fraser et al., 2022). V160 is located at the β3 in the SRCR domain, and the main chain of V160 forms antiparallel β-sheet interaction by hydrogen bonds. V160 is conserved between the TTSP family proteins (Ohno et al., 2021). The SRCR domain is located on the backside of the SP domain away from the active site (Fraser et al., 2022). Thus, the exact function of the SRCR domain remains unclear, but it is predicted to be necessary for protein–protein interactions and substrate recruitment by TTSPs (Yap et al., 2015). In contrast to the dominant model (C/C vs. [C/T + T/T]), no differences were found in the recessive model ([C/C + C/T] vs. T/T; Figure 2B). This may suggest that the V160 could not overcome the reduced function of M160 with respect to the substrate recognition and/or intermolecular autocleavage activation. We found that the V160M substitution of TMPRSS2 impairs not only the virus internalization on the plasma membrane but also the spike-mediated syncytium formation (Figures 3D–G). Even though the reduction is not remarkable, the V160M substitution delays the viral entry and may provide an opportunity for viral clearance at the mucosal surfaces by respiratory mucus, which traps pathogens that enter the airways. In addition, not only cell-free but also cell-to-cell virus transmission by the syncytium formation is important for the virus replication (Yu et al., 2022). The syncytium formation is frequently observed in the lungs of COVID-19 patients and is a defining pathological feature of COVID-19 patients who died as a direct consequence of SARS-CoV-2 infection (Bussani et al., 2020; Sanders et al., 2021). Although the detail mechanism remains unclear how the syncytium formation is involved in the COVID-19 disease progression, the decreased syncytium formation by the V160M substitution may lead to a reduction in the severity of COVID-19.

The T allele in rs12329760 *TMPRSS2* had no protective effect against development of severe COVID-19 symptoms. This lack of a protective effect is possibly due to other risk factors, such as the presence of other risk allele, the presence of underlying diseases, and the relatively older age of the patients with severe symptoms compared with those with nonsevere symptoms. Further investigations are needed to understand the contribution of each risk factor to COVID-19 disease progression.

## Data availability statement

The datasets presented in this study can be found in online repositories. The names of the repository/repositories and accession number(s) can be found at: https://www.ddbj.nig.ac.jp/, JGAS000562.

## Ethics statement

The studies involving human participants were reviewed and approved by The Medical Ethics Committee of University of Tsukuba. The patients/participants provided their written informed consent to participate in this study.

## Author contributions

TS, HKa, AK, and KY: conceived and designed the experiments. TS, HKa, SO, MH, TK, YO, and AK: performed the experiments, and TS, HKa, TK, AK, WM, and KY: analyzed the data. AH, YH, SH, YK, YA, KM, SC, HS, HKo, YO, RE, and HT: provided biological materials. TS, HKa, AK, YO, WM, and KY: wrote the manuscript. All authors contributed to the article and approved the submitted version.

## Funding

This work was supported in part by grants-in-aid from the Ministry of Health, Labour and Welfare Special Research Projects (20CA2055 to K.Y.) and the Ministry of Education, Culture, Sports, Science and Technology of Japan (19 J20101 to T.K. and 16H05192, 19H03475, and 22H02874 to A.K.); the Research Program on Emerging and Re-emerging Infectious Diseases (JP20fk0108076h0003 to A.K.); the Japan Program for Infectious Diseases Research and Infrastructure (JP20wm0325019h0001 to A.K.); the Japan Agency for Medical Research and Development, CREST and COI-NEXT from the Japan Science and Technology Agency (JPMJCR20H6 and JPMJPF2017 to A.K.); the Takeda Science Foundation (to A.K.); and the NOMURA Microbial Community Control Project of ERATO, Japanese Science and Technology Agency (to A.K.).

## Conflict of interest

The authors declare that the research was conducted in the absence of any commercial or financial relationships that could be construed as a potential conflict of interest.

## Publisher’s note

All claims expressed in this article are solely those of the authors and do not necessarily represent those of their affiliated organizations, or those of the publisher, the editors and the reviewers. Any product that may be evaluated in this article, or claim that may be made by its manufacturer, is not guaranteed or endorsed by the publisher.

## Footnotes

^1^ https://center6.umin.ac.jp/cgi-open-bin/ctr_e/ctr_view.cgi?recptno = R000047878

^2^ https://ldlink.nci.nih.gov/?tab=home

^3^ https://www.covid19-taskforce.jp/en/home/

## References

[ref1] Bertram S. Dijkman R. Habjan M. Heurich A. Gierer S. Glowacka I.. (2013). TMPRSS2 activates the human coronavirus 229E for Cathepsin-independent host cell entry and is expressed in viral target cells in the respiratory epithelium. J. Virol. 87, 6150–6160. doi: 10.1128/jvi.03372-12, PMID: 23536651 PMC3648130

[ref2] Böttcher E. Matrosovich T. Beyerle M. Klenk H.-D. Garten W. Matrosovich M. (2006). Proteolytic activation of influenza viruses by serine proteases TMPRSS2 and HAT from human airway epithelium. J. Virol. 80, 9896–9898. doi: 10.1128/JVI.01118-06, PMID: 16973594 PMC1617224

[ref3] Bugge T. H. Antalis T. M. Wu Q. (2009). Type II transmembrane serine proteases. J. Biol. Chem. 284, 23177–23181. doi: 10.1074/JBC.R109.021006, PMID: 19487698 PMC2749090

[ref4] Bussani R. Schneider E. Zentilin L. Collesi C. Ali H. Braga L.. (2020). Persistence of viral RNA, pneumocyte syncytia and thrombosis are hallmarks of advanced COVID-19 pathology. EBioMedicine 61:103104. doi: 10.1016/J.EBIOM.2020.103104, PMID: 33158808 PMC7677597

[ref5] Cheng Z. Zhou J. To K. K.-W. Chu H. Li C. Wang D.. (2015). Identification of TMPRSS2 as a susceptibility gene for severe 2009 pandemic a(H1N1) influenza and a(H7N9) influenza. J. Infect. Dis. 212, 1214–1221. doi: 10.1093/infdis/jiv246, PMID: 25904605 PMC7107393

[ref6] Ellinghaus D. Degenhardt F. Bujanda L. Buti M. Albillos A. Invernizzi P.. (2020). Genomewide association study of severe Covid-19 with respiratory failure. N. Engl. J. Med. 383, 1522–1534. doi: 10.1056/NEJMoa2020283, PMID: 32558485 PMC7315890

[ref7] FitzGerald L. M. Agalliu I. Johnson K. Miller M. A. Kwon E. M. Hurtado-Coll A.. (2008). Association of TMPRSS2-ERG gene fusion with clinical characteristics and outcomes: results from a population-based study of prostate cancer. BMC Cancer 8:230. doi: 10.1186/1471-2407-8-230, PMID: 18694509 PMC2519091

[ref8] Fraser B. J. Beldar S. Seitova A. Hutchinson A. Mannar D. Li Y.. (2022). Structure and activity of human TMPRSS2 protease implicated in SARS-CoV-2 activation. Nat. Chem. Biol. 18, 963–971. doi: 10.1038/s41589-022-01059-7, PMID: 35676539

[ref9] Gierer S. Bertram S. Kaup F. Wrensch F. Heurich A. Krämer-Kühl A.. (2013). The spike protein of the emerging betacoronavirus EMC uses a novel coronavirus receptor for entry, can be activated by TMPRSS2, and is targeted by neutralizing antibodies. J. Virol. 87, 5502–5511. doi: 10.1128/jvi.00128-13, PMID: 23468491 PMC3648152

[ref10] Guo Y. Kawaguchi A. Takeshita M. Sekiya T. Hirohama M. Yamashita A.. (2021). Potent mouse monoclonal antibodies that block SARS-CoV-2 infection. J. Biol. Chem. 296:100346. doi: 10.1016/j.jbc.2021.100346, PMID: 33524396 PMC7846482

[ref11] Hoffmann M. Kleine-Weber H. Schroeder S. Krüger N. Herrler T. Erichsen S.. (2020). SARS-CoV-2 cell entry depends on ACE2 and TMPRSS2 and is blocked by a clinically proven protease inhibitor. Cells 181, 271–280.e8. doi: 10.1016/j.cell.2020.02.052, PMID: 32142651 PMC7102627

[ref12] Iwasaki A. Grubaugh N. D. (2020). Why does Japan have so few cases of COVID-19? EMBO Mol. Med. 12:e12481. doi: 10.15252/emmm.202012481, PMID: 32275804 PMC7207161

[ref13] Lai C. C. Liu Y. H. Wang C. Y. Wang Y. H. Hsueh S. C. Yen M. Y.. (2020). Asymptomatic carrier state, acute respiratory disease, and pneumonia due to severe acute respiratory syndrome coronavirus 2 (SARS-CoV-2): facts and myths. J. Microbiol. Immunol. Infect. 53, 404–412. doi: 10.1016/J.JMII.2020.02.012, PMID: 32173241 PMC7128959

[ref14] Latini A. Agolini E. Novelli A. Borgiani P. Giannini R. Gravina P.. (2020). COVID-19 and genetic variants of protein involved in the SARS-CoV-2 entry into the host cells. Genes (Basel) 11, 1–8. doi: 10.3390/GENES11091010, PMID: 32867305 PMC7565048

[ref15] Matsuyama S. Nagata N. Shirato K. Kawase M. Takeda M. Taguchi F. (2010). Efficient activation of the severe acute respiratory syndrome coronavirus spike protein by the transmembrane protease TMPRSS2. J. Virol. 84, 12658–12664. doi: 10.1128/JVI.01542-10, PMID: 20926566 PMC3004351

[ref16] Namkoong H. Edahiro R. Takano T. Nishihara H. Shirai Y. Sonehara K.. (2022). DOCK2 is involved in the host genetics and biology of severe COVID-19. Nature 609, 754–760. doi: 10.1038/s41586-022-05163-5, PMID: 35940203 PMC9492544

[ref17] Ohno A. Maita N. Tabata T. Nagano H. Arita K. Ariyoshi M.. (2021). Crystal structure of inhibitor-bound human MSPL that can activate high pathogenic avian influenza. Life Sci. Alliance 4:e202000849. doi: 10.26508/LSA.202000849, PMID: 33820827 PMC8046417

[ref18] Pairo-Castineira E. Clohisey S. Klaric L. Bretherick A. D. Rawlik K. Pasko D.. (2021). Genetic mechanisms of critical illness in COVID-19. Nature 591, 92–98. doi: 10.1038/S41586-020-03065-Y 33307546

[ref19] Paniri A. Hosseini M. M. Akhavan-Niaki H. (2020). First comprehensive computational analysis of functional consequences of TMPRSS2 SNPs in susceptibility to SARS-CoV-2 among different populations. J. Biomol. Struct. Dyn. 39, 3576–3593. doi: 10.1080/07391102.2020.1767690, PMID: 32410502 PMC7284145

[ref20] Sanders D. W. Jumper C. C. Ackerman P. J. Bracha D. Donlic A. Kim H.. (2021). SARS-CoV-2 requires cholesterol for viral entry and pathological syncytia formation. elife 10, 1–47. doi: 10.7554/eLife.65962, PMID: 33890572 PMC8104966

[ref21] Schönfelder K. Breuckmann K. Elsner C. Dittmer U. Fistera D. Herbstreit F.. (2021). Transmembrane serine protease 2 polymorphisms and susceptibility to severe acute respiratory syndrome coronavirus type 2 infection: a German case-control study. Front. Genet. 12:585. doi: 10.3389/fgene.2021.667231, PMID: 33968142 PMC8097083

[ref22] Scully E. P. Haverfield J. Ursin R. L. Tannenbaum C. Klein S. L. (2020). Considering how biological sex impacts immune responses and COVID-19 outcomes. Nat. Rev. Immunol. 20, 442–447. doi: 10.1038/S41577-020-0348-8, PMID: 32528136 PMC7288618

[ref23] Yamamoto N. Bauer G. (2020). Apparent difference in fatalities between Central Europe and East Asia due to SARS-COV-2 and COVID-19: four hypotheses for possible explanation. Med. Hypotheses 144:110160. doi: 10.1016/j.mehy.2020.110160, PMID: 32795831 PMC7403102

[ref24] Yang J. Zheng Y. Gou X. Pu K. Chen Z. Guo Q.. (2020). Prevalence of comorbidities and its effects in patients infected with SARS-CoV-2: a systematic review and meta-analysis. Int. J. Infect. Dis. 94, 91–95. doi: 10.1016/J.IJID.2020.03.017, PMID: 32173574 PMC7194638

[ref25] Yap N. V. L. Whelan F. J. Bowdish D. M. E. Golding G. B. (2015). The evolution of the scavenger receptor cysteine-rich domain of the class a scavenger receptors. Front. Immunol. 6:342. doi: 10.3389/fimmu.2015.00342, PMID: 26217337 PMC4491621

[ref26] Yu S. Zheng X. Zhou B. Li J. Chen M. Deng R.. (2022). SARS-CoV-2 spike engagement of ACE2 primes S20 site cleavage and fusion initiation. Proc. Natl. Acad. Sci. U. S. A. 119:e2111199119. doi: 10.1073/pnas.2111199119, PMID: 34930824 PMC8740742

