# Supplementary material for: TMPRSS2 gene polymorphism common in East Asians confers decreased COVID-19 susceptibility
Source: Front Microbiol. 2022 Nov 30;13:943877. doi: 10.3389/fmicb.2022.943877 (PMC9748344; doi:10.3389/fmicb.2022.943877)
Supplement: Supplementary file 1 [file Data_Sheet_1.docx]

Supplementary Material

|  | Infected | Noninfected | *P* value |
| --- | --- | --- | --- |
| Number | 25 | 139 |  |
| Age, median (IQR), y | 34 (30-43) | 40 (31-48) | 0.14 |
| Male, n (%) | 9 (36) | 43 (30.9) | 0.64 |
| Body mass index, median (IQR), kg/m^2^ | 22.9 (21.6-25.3) | 22.1 (20.4-24.8) | 0.21 |
| Systolic arterial pressure, median (IQR), mmHg | 123 (112-128) | 117 (107.5-126) | 0.13 |
| Diastolic arterial pressure, median (IQR), mmHg | 76 (69-81) | 70 (60.5-79) | 0.03 |
| Mean arterial pressure, median (IQR), mmHg | 90.7 (86.3-96) | 86.7 (77.9-93) | 0.047 |
| Smoking, n (%) | 4 (16) | 49 (35.3) | 0.18 |
| BCG vaccination, n (%) | 22 (88) | 114 (82) | 0.91 |
| Hypertension, n (%) | 2 (8) | 8 (5.8) | 0.65 |
| Diabetes mellitus, n (%) | 1 (4) | 0 (0) | 0.15 |
| Respiratory disease, n (%) | 3 (12) | 9 (6.5) | 0.11 |
| Cardiac disease, n (%) | 0 (0) | 3 (2.2) | 1 |
| Allergy, n (%) | 4 (16) | 17 (12.2) | 0.53 |
| Gynecologic disease, n (%) | 0 (0) | 2 (2.1) | 1 |
| Thyroid disease, n (%) | 0 (0) | 3 (2.2) | 1 |
| Analgesic, n (%) | 0 (0) | 7 (5) | 0.6 |
| Malignancy, n (%) | 0 (0) | 0 (0) |  |
| Chronic kidney disease, n (%) | 0 (0) | 0 (0) |  |
|  |  |  |  |
| BCG, bacille Calmette-Guérin; IQR, interquartile range | | | |

**Supplementary Table 1: Clinical characteristics of t****he infected and noninfected participants**

All continuous characteristics are described as the medians (IQRs), and categorical characteristics are described as numbers (%). Significant differences of continuous characteristics between infected individuals and non-infected individuals were determined by the Mann–Whitney U test. Categorical characteristics comparison was performed with Fisher’s exact test.

|  | Infected | Noninfected | *P* value |
| --- | --- | --- | --- |
| White blood cells, median (IQR), 10^3^/μL | 6.7 (5.6-7.8) | 5.8 (5-6.9) | 0.06 |
| Neutrophils, median (IQR), % | 62.9 (58.4-67.9) | 59.7 (53.4-66.3) | 0.19 |
| Lymphocytes, median (IQR), % | 28.1 (25.8-34.2) | 31.6 (26.5-37.5) | 0.14 |
| Hemoglobin, median (IQR), g/dL | 14 (12.8-15.4) | 13.6 (12.6-14.8) | 0.41 |
| Hematocrit, median (IQR), % | 41.2 (39.1-45.6) | 40.3 (37.8-43.9) | 0.25 |
| Platelets, median (IQR), 10^3^/μL | 260 (238-358) | 263 (229-300) | 0.41 |
| Total protein, median (IQR), g/dL | 7.6 (7.4-7.7) | 7.3 (6.9-7.6) | 0.005 |
| Albumin, median (IQR), g/dL | 4.7 (4.5-4.9) | 4.5 (4.3-4.8) | 0.18 |
| AST, median (IQR), U/L | 19 (16-24) | 20 (17-23) | 0.43 |
| ALT, median (IQR), U/L | 14 (12-19) | 16 (11.5-22) | 0.58 |
| ALP IFCC, median (IQR), U/L | 59 (53-81) | 62 (54-75.5) | 0.74 |
| LDH IFCC, median (IQR), U/L | 161 (146-176) | 162 (150-177.5) | 0.67 |
| γGTP, median (IQR), U/L | 19 (13-22) | 19 (14-28) | 0.32 |
| Sodium, median (IQR), mEq/L | 141 (140-142) | 141 (140-142) | 0.52 |
| Potassium, median (IQR), mEq/L | 4 (3.8-4.2) | 3.9 (3.7-4.2) | 0.66 |
| Blood urea nitrogen, median (IQR), mg/dL | 13.6 (10.9-15.5) | 12.8 (11-15) | 0.86 |
| Serum creatinine, median (IQR), mg/dL | 0.66 (0.58-0.75) | 0.69 (0.6-0.83) | 0.17 |
| eGFR, median (IQR), mL/min/1.73m^2^ | 87.4 (79.6-111.8) | 82.8 (73.6-90.8) | 0.02 |
| Serum uric acid, median (IQR), mg/dL | 4.6 (3.7-6.3) | 4.5 (3.7-5.4) | 0.75 |
| IgG, median (IQR), mg/dL | 1314 (1165-1487) | 1196 (1072-1358) | 0.03 |
| Glycemia, median (IQR), mg/dL | 90 (83-112) | 96 (86-114.5) | 0.49 |
| HemoglobinA1c, median (IQR), % | 5.4 (5.3-5.6) | 5.5 (5.3-5.7) | 0.71 |
| Glycoalbumin, median (IQR), % | 12.8 (12-13.2) | 13.1 (12.5-13.8) | 0.07 |
| D-dimer, median (IQR), μg/mL | 0.6 (0.5-0.6) | 0.5 (0.5-0.6) | 0.35 |
| CRP, median (IQR), mg/dL | 0 (0-0.04) | 0 (0-0.04) | 0.61 |
| Dipstick proteinuria, n (%) | 1 (4) | 4 (2.9) | 0.64 |
| Dipstick hematuria, n (%) | 3 (12) | 13 (9.4) | 0.81 |
| Urinary protein/Urinary creatinine, median (IQR) | 0.04 (0.03-0.06) | 0.04 (0.03-0.05) | 0.61 |
| Serum ACE, median (IQR), ng/mL | 1.18 (0.72-1.69) | 1.22 (0.86-2.01) | 0.34 |

**Supplementary Table 2: Laboratory findings for infected and noninfected participants**

All continuous characteristics are described as the medians (IQRs), and categorical characteristics are described as numbers (%). Significant differences of continuous characteristics between infected individuals and non-infected individuals were determined by the Mann–Whitney U test. Categorical characteristics comparison was performed with Fisher’s exact test.

| Cytokine/Chemokine  (pg/ml) | Infected | Noninfected | *P* value |
| --- | --- | --- | --- |
| sCD40L | 6067.1 (4759.9-8276.4) | 7701.2 (5687.0-9845.1) | 0.01 |
| EGF | 82.3 (41.4-98.3) | 105.4 (57.0-153.3) | 0.13 |
| Eotaxin | 74.9 (64.2-91.2) | 92.7 (72.0-110.1) | 0.01 |
| FGF-2 | 46.8 (3.8-59.3) | 20.1 (0-40.3) | 0.14 |
| FLT3L | 7.5 (2.3-11.5) | 13.1 (6.8-22.8) | 0.008 |
| Fractalkine | 116.5 (36.6-180.8) | 111.7 (69.6-223.5) | 0.47 |
| G-CSF | 5.5 (0-26.6) | 8.2 (0-18.9) | 0.83 |
| GM-CSF | N.D. | N.D. | 0.60 |
| GROα/CXCL1 | 19.5 (11.8-29.5) | 17.2 (12.9-21.4) | 0.56 |
| IFN-α2 | 0 (0-20.2) | 7.8 (0-21.6) | 0.26 |
| IFN-γ | 0 (0-0.1) | 0 (0-6.3) | 0.10 |
| IL-1α | 1.1 (0-6.9) | 2.6 (0-10.5) | 0.43 |
| IL-1β | 6.6 (0-11.7) | 4.2 (0-11.0) | 0.92 |
| IL-1RA | 3.0 (2.3-5.4) | 2.9 (1.5-6.1) | 0.43 |
| IL-2 | N.D. | N.D. |  |
| IL-3 | N.D. | N.D. |  |
| IL-4 | 0 (0-0.6) | 0.1 (0-1.2) | 0.14 |
| IL-5 | 3.7 (2.9-7.6) | 2.9 (1.3-7.7) | 0.31 |
| IL-6 | 0 (0-0.3) | 0 (0-0.5) | 0.17 |
| IL-7 | 5.8 (3.5-12.3) | 4.5 (2.8-6.2) | 0.13 |
| IL-8 | 5.1 (4.0-6.6) | 5.4 (3.9-7.0) | 0.59 |
| IL-9 | 3.3 (0-24.7) | 7.6 (0-29.8) | 0.44 |
| IL-10 | N.D. | N.D. | 0.47 |
| IL-12 p40 | 15.3 (0.3-30.6) | 16.4 (4.2-29.9) | 0.61 |
| IL-12 p70 | 0 (0-1.0) | 0 (0-0.9) | 0.61 |
| IL-13 | 38.0 (6.3-71.4) | 51.9 (21.7-107.0) | 0.13 |
| IL-15 | 4.7 (4.0-7.7) | 6.4 (3.8-9.0) | 0.37 |
| IL-17A | 0 (0-8.5) | 0 (0-2.6) | 0.10 |
| IL-17E/IL-25 | 342.2 (235.8-709.4) | 320.9 (214.2-527.5) | 0.55 |
| IL-17F | N.D. | N.D. | 0.89 |
| IL-18 | 9.4 (5.8-18.4) | 11.1 (5.2-16.8) | 0.66 |
| IL-22 | N.D. | N.D. | 0.39 |
| IL-27 | 1092.9 (767.4-1573.4) | 1246.5 (933.2-1767.0) | 0.23 |
| IP-10 | 171.3 (114.1-198.1) | 144.4 (118.5-190.9) | 0.76 |
| MCP-1 | 382.9 (293.1-483.1) | 400.6 (330.1-464.6) | 0.66 |
| MCP-3 | 19.3 (0.7-27.0) | 20.0 (7.0-41.6) | 0.45 |
| M-CSF | 10.4 (0-36.7) | 26.4 (0-55.5) | 0.17 |
| MDC/CCL22 | 714.4 (558.2-809.4) | 652.3 (526.2-826.3) | 0.49 |
| MIG/CXCL9 | 848.4 (618.2-989.0) | 907.2 (662.2-1165.6) | 0.44 |
| MIP-1α | 4.1 (0-30.8) | 13.0 (0-33.0) | 0.33 |
| MIP-1β | 29.5 (21.5-32.5) | 25.9 (17.8-37.1) | 0.54 |
| PDGF-AA | 4711.2 (3652.9-5513.1) | 4460.5 (3506.6-5480.0) | 0.83 |
| PDGF-AB/BB | 30587.9 (26942.8-32858.8) | 30250.9 (26674.0-34089.0) | 0.99 |

**Supplementary Table 3: Normal physiological levels of serum cytokines and chemokines of the infected and noninfected participants**

**Supplementary Table 3 continued**

| Cytokine/Chemokine  (pg/ml) | Infected | Noninfected | *P* value |
| --- | --- | --- | --- |
| TGF-α | 3.6 (2.9-7.0) | 3.4 (1.6-5.3) | 0.16 |
| TNF-α | 12.1 (8.5-28.0) | 16.1 (10.8-31.9) | 0.16 |
| TNF-β | 8.6 (3.2-15.8) | 6.7 (3.2-17.4) | 0.94 |
| VEGF-A | 316.9 (182.4-381.5) | 301.6 (141.8-446.3) | 0.77 |
| RANTES | N.D. | N.D. |  |

Significant differences of continuous characteristics between infected individuals and non-infected individuals were determined by the Mann–Whitney U test.

|  | Infected | Noninfected | *P* value |
| --- | --- | --- | --- |
| None | 0 (0%) | 15 (10.8%) | 0.22 |
| Extremely light contact | 3 (15%) | 26 (18.7%) | 1 |
| Light contact  (eg, vital signs measurement) | 5 (25%) | 62 (44.6%) | 0.15 |
| Tight contact (eg, repositioning) | 12 (60%) | 39 (28.1%) | 0.01 |
| Oral cavity and respiratory tract suction | 10 (50%) | 26 (18.7%) | 0.004 |
| No recollection | 1 (5%) | 9 (6.5%) | 1 |

**Supplementary Table 4: Medical care given to COVID-19 patients**

Categorical characteristics comparison was performed with Fisher’s exact test.

|  |  | Infected | | | | |  | Noninfected | | | | |  |
| --- | --- | --- | --- | --- | --- | --- | --- | --- | --- | --- | --- | --- | --- |
| Rate of implementation | | 100% | 75% | 50% | 25% | 0% |  | 100% | 75% | 50% | 25% | 0% | *P* value |
| Personal protective equipment | Hand-washing | 4  (21.1%) | 10  (52.6%) | 5  (26.3%) | 0  (0%) | 0  (0%) |  | 40  (28.8%) | 75  (54%) | 21  (15.1%) | 1  (0.7%) | 2  (1.4%) | 0.66 |
|  | Surgical mask | 20  (100%) | 0  (0%) | 0  (0%) | 0  (0%) | 0  (0%) |  | 117  (84.2%) | 10  (7.2%) | 1  (0.7%) | 2  (1.4%) | 9  (6.5%) | 0.59 |
|  | N95 mask | 4  (20%) | 1  (5%) | 1  (5%) | 1  (5%) | 13  (65%) |  | 25  (18%) | 6  (4.3%) | 4  (2.9%) | 3  (2.2%) | 101  (72.7%) | 0.53 |
|  | Eye protection | 9  (45%) | 4  (20%) | 3  (15%) | 2  (10%) | 2  (10%) |  | 51  (36.7%) | 8  (5.8%) | 13  (9.4%) | 8  (5.8%) | 59  (42.4%) | 0.01 |
|  | Medical gown | 6  (30%) | 3  (15%) | 2  (10%) | 2  (10%) | 7  (35%) |  | 38  (27.5%) | 6  (4.3%) | 11  (8%) | 7  (5.1%) | 76  (55.1%) | 0.13 |
|  | Medical gloves | 14  (70%) | 3  (15%) | 1  (5%) | 0  (0%) | 2  (10%) |  | 61  (43.9%) | 19  (13.7%) | 12  (8.6%) | 10  (7.2%) | 37  (26.6%) | 0.22 |

**Supplementary Table 5: Rates of implementation of personal protective measures by infected and noninfected participants**

Categorical characteristics comparison was performed with Fisher’s exact test.
